# The Efflux Mechanism of Fraxetin-*O*-Glucuronides in UGT1A9-Transfected HeLa Cells: Identification of Multidrug Resistance-Associated Proteins 3 and 4 (MRP3/4) as the Important Contributors

**DOI:** 10.3389/fphar.2019.00496

**Published:** 2019-05-07

**Authors:** Zifei Qin, Beibei Zhang, Jing Yang, Shishi Li, Jinjin Xu, Zhihong Yao, Xiaojian Zhang, Frank J. Gonzalez, Xinsheng Yao

**Affiliations:** ^1^Department of Pharmacy, First Affiliated Hospital of Zhengzhou University, Zhengzhou, China; ^2^Henan Key Laboratory of Precision Clinical Pharmacy, Zhengzhou University, Zhengzhou, China; ^3^College of Pharmacy, Jinan University, Guangzhou, China; ^4^Guangdong Province Key Laboratory of Pharmacodynamic Constituents of TCM and New Drugs Research, Jinan University, Guangzhou, China; ^5^Laboratory of Metabolism, Center for Cancer Research, National Cancer Institute, National Institutes of Health, Bethesda, MD, United States

**Keywords:** fraxetin, UGT1A9, glucuronidation, efflux transporters, HeLa1A9 cells, MRPs

## Abstract

Fraxetin, a natural compound present in many dietary supplements and herbs, is useful in the treatment of acute bacillary dysentery and type 2 diabetes. Previously, several metabolic studies have revealed extensive first-pass metabolism causing formation of fraxetin-*O*-glucuronides (G1 and G2), resulting in poor bioavailability of fraxetin. Active transport processes play an important role in the excretion of fraxetin-*O*-glucuronides. Nevertheless, the transporters involved are yet to be elucidated. In this study, we aimed to determine the active efflux transporters, including breast cancer resistance protein (BCRP) and multidrug resistance-associated proteins (MRPs), involved in the excretion of fraxetin-*O*-glucuronides. A chemical inhibitor, MK571 (5 and 20 μM), a pan-MRP inhibitor, led to a significant decrease in excreted G1 (maximal 59.1%) and G2 levels (maximal 42.4%), whereas Ko143 (5 and 20 μM), a selective BCRP inhibitor, caused moderate downregulation of excreted G1 (maximal 29.4%) and G2 (maximal 28.5%). Furthermore, MRP3 silencing resulted in a marked decrease of excretion rates (by 29.1% for G1 and by 21.1% for G2) and of fraction metabolized (*f*_met_; by 24.1% for G1 and by 18.6% for G2). Similar results, i.e., a significant reduction in excretion rates (by 34.8% for G1 and by 32.3% for G2) and in *f*_met_ (by 22.7% for G1 and by 23.1% for G2) were obtained when MRP4 was partially silenced. No obvious modifications in the excretion rates, intracellular levels, and *f*_met_ values of glucuronides were observed after short hairpin RNA (shRNA)-mediated silencing of transporters BCRP and MRP1. Taken together, our results indicate that MRP3 and MRP4 contribute more to the excretion of fraxetin-*O*-glucuronides than the other transporters do.

## Introduction

Fraxetin is a natural coumarin analog that is widely distributed in many functional foods and dietary supplements (Jyotshna et al., 2017). It has also been demonstrated to be effective as a therapeutic agent with multiple activities, such as antioxidant, antibacterial, hypoglycemic, antiosteoporosis, and antiplatelet activities (Kuo et al., 2006; Murali et al., 2013; Wang et al., 2014; Feng et al., 2016; Zaragoza et al., 2016). In addition, it was identified as a cystathionine β-synthase inhibitor (*IC*_50_ = 134 μM) that plays a critical role in human sulfur metabolism (Thorson et al., 2013). Fraxetin has a significantly lower half-maximal inhibitory concentration (*IC*_50_) than acarbose does (a well-known α-glucosidase inhibitor) (Milella et al., 2016), in agreement with the finding that fraxetin can control glucose metabolism in the liver and kidneys, resulting in a substantial reduction in the risk of type 2 diabetes (Murali et al., 2013). Recently, it was reported that fraxetin may serve as a promising candidate drug against type 2 diabetes through antioxidative mechanisms (Yao et al., 2018). These significant biological properties led to increased interest in the *in vivo* metabolic fate and pharmacokinetic characteristics of fraxetin.

Sampling of rat urine after oral administration of fraxin (fraxetin-7-*O*-β-glucoside) showed that fraxin can be primarily hydrolyzed to fraxetin by the microflora abundant in the intestine (Yasuda et al., 2006). The maximal plasma concentration and half-life of fraxetin are 2250 ng/mL (∼11 μM) and 11 h after oral administration of fraxin (50 mg/kg) in rats, respectively (Wang H. et al., 2016). In addition, fraxetin has pharmacokinetic parameters similar to those of fraxin after treatment of rodents with *Cortex Fraxini* extracts (including 60 mg/kg fraxin and 40 mg/kg fraxetin) (Wang et al., 2017). In contrast to the hydrolysis reaction in rodents, a novel enzyme named scopoletin 8-hydroxylase catalyzes hydroxylation at the C-8 position of scopoletin, leading to fraxetin production in plants (Siwinska et al., 2018). These findings indicate that fraxetin and fraxin are both the most abundant components in the circulatory system after oral administration of fraxin or fraxin-containing foods and herbs, and also suggest that the *in vivo* pharmacological activities of fraxin may be attributed to fraxin and fraxetin.

On the other hand, fraxetin is a natural catechol-containing coumarin that can be readily metabolized by phase II enzymes. In fact, fraxetin-7-*O*-glucuronide and fraxetin-7-*O*-sulfate have both been isolated from rat urine after oral administration of fraxin (Yasuda et al., 2006). Subsequently, UDP-glucuronosyltransferase 1A9 (UGT1A9) was identified as the major isoform responsible for fraxetin-*O*-glucuronidation reactions (*CL*_int_ values between 42 and 300 μL/min per milligram of protein for fraxetin-7-*O*-glucuronide and fraxetin-8-*O*-glucuronide, respectively) (Xia et al., 2014). Traditionally, it has been noted that two processes are necessary for intracellular to extracellular glucuronide transport, namely glucuronide formation and glucuronide excretion, both of which influence the pharmacokinetics of drugs or natural compounds (Wu et al., 2011; Sun et al., 2015). Because glucuronides cannot penetration cell membranes due to high hydrophilicity, active transport of glucuronides by efflux transporters is necessary; the transporters include mainly *breast cancer resistance protein* (BCRP) and *multidrug resistance–associated proteins* (MRPs). The efflux transporters involved in the disposition of fraxetin-*O*-glucuronides have not been identified yet.

In this study, we applied an established tool, HeLa-UGT1A9 cells, to determine the efflux transport mechanism of fraxetin-*O*-glucuronides (Wei et al., 2013). The use of HeLa1A9 cells for glucuronide transport studies was more advantageous compared to the use of membrane vesicles for overexpression of a transporter. This is because (1) purified glucuronides (whose commercial availability is poor) are not required as glucuronides and are produced within HeLa1A9 cells from the aglycone via the action of UGT1A9 (Wei et al., 2013); (2) several efflux transporters including BCRP, MRP1, MRP3, and MRP4 are all expressed in HeLa cells (Quan et al., 2015). In addition, HeLa1A9 cells are more advantageous than Caco-2 cells, because Caco-2 cells can express numerous drug-metabolizing enzymes including cytochrome P450 (CYPs), UGTs, and sulfate transferases (SULTs), resulting in complex metabolic pathways of drugs (Quan et al., 2015). Moreover, the evaluation of glucuronide transport would be seriously influenced by simultaneous transport of other types of metabolites (e.g., fraxetin-*O*-sulfates). In addition, this HeLa1A9 cell model had been successfully applied to evaluate the glucuronidation-transport interplay (Jiang et al., 2011; Wu et al., 2012; Wei et al., 2013; Passi et al., 2016). Hence, we used HeLa1A9 cell model to explore the mechanism through which transporters BCRP and MRP affected fraxetin disposition. These results should contribute to improved understanding on the pharmacokinetics behavior of fraxetin.

## Materials and Methods

### Materials

Human UGT1A9-overexpressing HeLa cells (HeLa1A9 cells) were established as described previously (Jiang et al., 2011), and were provided by Prof. Baojian Wu who works in Jinan University in Guangzhou of China. Alamethicin, D-saccharic-1,4-lactone, magnesium chloride (MgCl_2_), MK571, Ko143, propofol, and uridine diphosphate glucuronic acid (UDPGA) were purchased from Sigma-Aldrich (St Louis, MO, United States), whereas propofol-*O*-glucuronide from Toronto Research Chemicals (North York, ON, Canada). Fraxetin was provided by Guangzhou Fans Biotechnology Co., Ltd., (Guangzhou, China), and human UGT1A9 was purchased from Corning Biosciences (New York, NY, United States). UGT1A9 antibody was purchased from BD Biosciences (Woburn, MA, United States). The anti-BCRP, anti-MRP1, anti-MRP2, anti-MRP3, and anti-MRP4 antibodies were purchased from OriGene Technologies (Rockville, MD, United States). All other chemicals and reagents (analytical grade or better) were commercially available.

### Preparation of a HeLa1A9 Cell Lysate

HeLa1A9 cells were grown in 10 cm dishes for 3 to 4 days and then were washed and harvested in 50 mM Tris buffer (*pH* = 7.4). The collected cells were sonicated using a tight-fitting Dounce homogenizer in an ice-cold water bath (4°C). Due to the thermal stability, the glucuronidation activity of UGT1A9 was not affected during sonication (Fujiwara et al., 2007). Next, the cell lysates were centrifuged at 4°C (13800 × *g* for 10 min). The supernatant to be used in the UGT glucuronidation activity assay was collected, and its protein concentration was measured by the bicinchoninic acid assay (BCA; Beyotime, Shanghai, China).

### Glucuronidation Activity Assays

The incubation system was slightly modified on the basis of a previously published article (Qin et al., 2018a). We mixed UGT1A9 (1.0 mg/mL) or HeLa1A9 cell lysates (2.1 mg/mL), alamethicin (20 μg/mL), D-saccharic-1,4-lactone (4.4 mM), MgCl_2_ (4 mM), and different concentrations of propofol (5–400 μM) or fraxetin (0.5–80 μM) in 50 mM Tris buffer (*pH* = 7.4). After preincubation at 37°C for 5 min, UDPGA (3.5 mM) was added to the reaction system, and the mixture was incubated for another 30 min. These reactions were terminated by the addition of an equal volume of ice-cold acetonitrile. The mixed samples were centrifuged at 13800 × *g* for 10 min, and the supernatant was analyzed by ultra-high-performance liquid chromatography (UHPLC). All the experiments were conducted in triplicate.

In addition, niflumic acid, magnolol, and androsterone were all employed as selective inhibitors for UGT1A9 (Xia et al., 2014; Yao et al., 2017). Fraxetin (4 and 20 μM) was incubated in the absence or presence of magnolol (1 μM), niflumic acid (10 μM), and androsterone (10 μM) to investigate the formation rates of fraxetin-*O*-glucuronides. Moreover, to better understand the effects of a chemical inhibitor, Ko143 (a selective BCRP inhibitor) and MK571 (a pan-MRP inhibitor), on the glucuronidation activity of fraxetin, the chemical inhibitors were included in the incubation mixture as reported previously (Qin et al., 2018a). Other detailed operations were the same as described above.

### Excretion Experiments

The HeLa1A9 cells were pretreated and incubated with fraxetin solutions as reported previously (Jiang et al., 2011). Before assays, the HeLa1A9 cells were washed twice with prewarmed (37°C) Hank’s balanced salt solution (HBSS; *pH* = 7.4). After that, fraxetin (10 μM) dissolved in 2 mL of HBSS was incubated with the HeLa1A9 cells. At each time point (30, 60, 90, and 120 min), 200 μL of the culture supernatant from each well was collected as samples, and an equal volume of a loading media was used to replenish each well. Next, the collected samples were each mixed with 100 μL ice-cold acetonitrile. The supernatants were subjected to UHPLC analysis after centrifugation (10 min at 13800 × *g*). In all glucuronide excretion experiments, cell death was not observed, which indicated that fraxetin had no significant toxicity toward HeLa1A9 cells and the excretion of glucuronides could be well evaluated.

In addition, Ko143 (5 and 20 μM) and MK571 (5 and 20 μM), as the specific chemical inhibitors of BCRP and MRPs (Quan et al., 2015; Qin et al., 2018a), respectively, were separately dissolved in an HBSS microtube containing fraxetin (10 μM) to investigate the effects of chemical inhibitors of efflux transporters. During the experiments, fraxetin had no significant toxicity toward HeLa1A9 cells within the experimental concentration range, and the glucuronide excretion could be evaluated well.

### Cloned Short Hairpin RNA (shRNA)-Mediated Partial Silencing of BCRP and MRPs

In glucuronide excretion assays, biological inhibition mainly including shRNA-mediated partial silencing of BCRP and MRPs in HeLa cells was usually performed to evaluate the role of each efflux transporter (Quan et al., 2015; Sun et al., 2015; Qin et al., 2018a). Established shRNA_BCRP, shRNA_MRP1, shRNA_MRP3, and shRNA_MRP4 plasmids were transiently transfected into HeLa1A9 cells to develop the stably shRNA-transfected cell lines, named HeLa1A9-BCRP-shRNA cells, HeLa1A9-MRP1-shRNA, HeLa1A9-MRP3-shRNA, and HeLa1A9-MRP4-shRNA cells, respectively. After transfection for 2 days, these engineered HeLa1A9 cells were subjected to fraxetin-*O*-glucuronide excretion experiments. The control scrambled shRNA served as a negative control.

### Data Analysis

The enzyme kinetic parameters were obtained by fitting appropriate kinetic models to the experimental data in GraphPad Prism V5 software (San Diego, CA, United States). The best model was selected based on visual inspection of the Eadie–Hofstee plots (Qin et al., 2018b). In brief, if the Eadie–Hofstee plot was linear, formation rates (*V*) of glucuronides at different substrate concentrations (C) were fitted to the standard equations (1):

(1) $$\text{V}=\frac{{\text{V}}_{\text{max}}\times [\text{S}]}{{\text{K}}_{\text{m}}+[\text{S}]}$$

Where K_m_ is the Michaelis–Menten constant, and V_max_ is the maximum rate of glucuronidation. The intrinsic clearance (CL_int_) was computed as V_max_/K_m_.

In addition, the excretion rate of intracellular glucuronides was calculated using Equ. 2. Fraction metabolized or the *f*_met_ value was defined as the fraction of a dose metabolized based on Equ. 3. The *f*_met_ value was regarded as the more appropriate parameter to reflect the extent of drug metabolism in intact cells in the presence of a transporter–enzyme interplay.

(2) $$\mathrm{Excretion rate}=V\frac{\mathrm{dC}(\mathrm{excretedglucuronide})}{\mathrm{dt}}$$

(3) $$f_{\mathrm{met}}=\frac{\mathrm{excreted glucuronide}+\mathrm{intracellular glucuronide}}{\mathrm{dosed aglycone}}$$

where V is the volume of the incubation medium, C is the excreted cumulative concentration of glucuronides, and *t* denotes the incubation time. Here, dC(excreted glucuronide)/dt describes the changes in glucuronide levels with time.

### Quantification of Fraxetin and Fraxetin-*O*-Glucuronides

Due to the lack of a reference standard, the quantification of fraxetin-*O*-glucuronides was based on the standard curve of the parent compound (fraxetin) according to the assumption that the parent compound and its glucuronides have closely similar UV absorbance maxima (Qin et al., 2018b). Hence, fraxetin and fraxetin-*O*-glucuronides were separated via an Acquity^TM^ UHPLC I-Class system (Waters Corporation, Manchester, United Kingdom) equipped with a BEH C18 column (2.1 mm × 50 mm, 1.7 μm, Waters, Ireland, Part NO. 186002350) at 35 °C. The mobile phase consisted of water (A) and acetonitrile (B) (both including 0.1% formic acid, V/V) at a flow rate of 0.4 mL/min. The gradient elution program was 2–2% B from minute 0 to 0.5, 2–22% B from minute 0.5 to 1.5, 22–40% B from minute 1.5 to 2.5, 40–90% B from minute 2.5 to 3.5, 90–2% B from minute 3.5 to 4.0, keeping 2% B from minute 4.0 to 4.5. The detection wavelength was 338 nm. The limit of detection (LOD) and limit of quantification (LOQ) were calculated as 3-fold and 10-fold of the ratio of signal-to-noise (S/N), respectively. The LOQ for fraxetin was 0.01 μM. Calibration curves were constructed by plotting the peak areas (Y) versus the concentrations (X) of fraxetin (analyte) by means of the 1/x^2^ weighting factor. Acceptable linear correlation (*Y* = 5038.6×) was confirmed via a correlation coefficient (*r*^2^) of 0.9996. The linear range was 0.01–10 μM. The accuracy and precision of the intra-day and inter-day measurements were both less than 4.4%.

The UHPLC system was coupled to a hybrid quadrupole orthogonal time-of-flight tandem mass spectrometer (SYNAPT^TM^ G2 HDMS, Waters, Manchester, United Kingdom) with electrospray ionization (ESI). The operating parameters were as follows: capillary voltage, 3 kV (ESI+); sample cone voltage, 35 V; extraction cone voltage, 4 V; source temperature, 100°C; desolvation temperature, 300°C; cone gas flow, 50 L/h; and desolvation gas flow, 800 L/h. The full scan mass range was 50–1500 Da. The method employed lock spray with leucine enkephalin (*m/z* 556.2771 in positive ion mode) to ensure mass accuracy.

### Western Blotting Assays

The experimental procedure for western blotting was similar to that of a published study (Quan et al., 2015). In brief, the HeLa1A9 cell lysate was subjected to sodium dodecyl sulfate (SDS)-polyacrylamide gel electrophoresis. The resulting cell lysate (40 μg total protein) was analyzed by SDS-polyacrylamide gel electrophoresis (in 8% acrylamide gels) and transferred onto polyvinylidene difluoride membranes (Millipore, Bedford, MA, United States). Blots were probed with anti-UGT1A9 (1:500 dilution), anti-BCRP (1:500 dilution), anti-MRP1 (1:250 dilution), anti-MRP2 (1:500 dilution), anti-MRP3 (1:500 dilution), and anti-MRP4 (1:1000 dilution) antibodies followed by a horseradish peroxidase-conjugated rabbit anti-goat IgG antibody (1:4000 dilution; Santa Cruz Biotechnology, Dallas, TX, United States). Protein bands were detected by enhanced chemiluminescence (ECL), and band intensities were measured by densitometry using the Quantity One software.

### Statistical Analysis

Mean differences between treatment and control groups were analyzed by Student’s *t*-test. Data were expressed as mean ± standard deviation (SD; *n* = 3), and the level of significance was set to *p* < 0.05 (^∗^) or *p* < 0.01 (^∗∗^) or *p* < 0.001 (^∗∗∗^).

**Table 1:** Kinetic parameters of propofol-*O*-glucuronidation, fraxetin-8-*O*-glucuronidation (G1), and fraxetin-7-*O*-glucuronidation (G2) by the UGT1A9 enzyme and by the HeLa1A9 cell lysate supplemented with uridine diphosphate glucuronic acid (UDPGA).

Substrate	Metabolite	Enzyme	*V*_max_ (pmol/min/mg)	*K*_m_ (μM)	*CL*_int_ [μL/(min.mg)]	Model
propofol	propofol-G	UGT1A9	61 ± 2	53 ± 6	1.2 ± 0.1	MM
propofol	propofol-G	HeLa1A9 cell lysate	3.2 ± 0.2 (^aaa^)	59 ± 13	0.10 ± 0.01 (^aaa^)	MM
fraxetin	G1	UGT1A9	2920 ± 160	16 ± 2	180 ± 28	MM
fraxetin	G2	UGT1A9	1440 ± 61	10 ± 1	150 ± 20	MM
fraxetin	G1	HeLa1A9 cell lysate	110 ± 5 (^bbb^)	13 ± 2	9 ± 1 (^bbb^)	MM
fraxetin	G2	HeLa1A9 cell lysate	42 ± 2 (^ccc^)	8 ± 1	5 ± 1 (^ccc^)	MM

*Data are presented as mean ± SD. ^aaa,bbb,ccc^p < 0.001 as compared with the parameters of propofol-O-glucuronidation and G1 and G2 formation by UGT1A9, respectively. Propofol-G, G1, and G2 mean propofol-O-glucuronide, fraxetin-8-O-glucuronide, and fraxetin-7-O-glucuronide, respectively. V_max_, maximal velocity; K_m_, Michaelis constant; CL_int_, intrinsic clearance. MM, Michaelis–Menten model.*

## Results

### Functional Validation of the HeLa1A9 Cell Model

**FIGURE 1:**
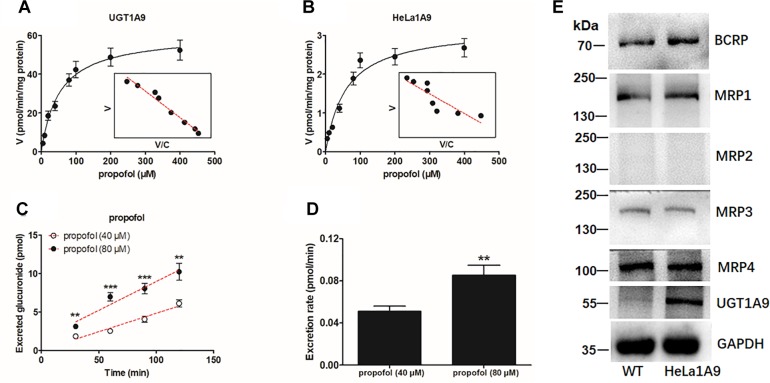
UGT1A9-mediated propofol-*O*-glucuronidation, excretion of propofol-*O*-glucuronide in HeLa1A9 cells, and expression of UGT1A9 and efflux transporters in HeLa1A9 cells. **(A)** Kinetic profiles for the glucuronidation of propofol (5–400 μM) by UGT1A9. **(B)** kinetic profiles for the glucuronidation of propofol (5–400 μM) by the HeLa1A9 cell lysate. In each panel, the inset shows the corresponding Eadie–Hofstee plot. **(C)** Excreted propofol-*O*-glucuronide in the extracellular solution at two concentrations of propofol (40 and 80 μM). **(D)** excretion rates of propofol-*O*-glucuronide at different concentrations (40 and 80 μM). **(E)** protein expression of transporters UGT1A9, BCRP, and four transporters of the MRP family in HeLa and HeLa1A9 cells. Data are presented as mean ± SD. ^∗^*p* < 0.05, ^∗∗^*p* < 0.01, or ^∗∗∗^*p* < 0.001 as compared with propofol (40 μM).

As mentioned above, the HeLa1A9 cell line was established as described previously (Jiang et al., 2011). In this study, to validate the function of UGT1A9 in HeLa cells, a well-accepted probe substrate of UGT1A9, propofol, was applied to evaluate the glucuronidation activity (Hong et al., 2017). Obviously, propofol-*O*-glucuronidation manifested the classical Michaelis–Menten kinetics of UGT1A9 (Figure 1A) and in the HeLa1A9 cell lysate (Figure 1B), and they had similar *K_m_* values (Table 1). In addition, propofol-*O*-glucuronide was produced and excreted into the extracellular solution after incubation of propofol (40 and 80 μM, close to the *K*_m_ values), with HeLa1A9 cells showing a linear increase within 120 min (Figure 1C). Besides, the excretion rates of propofol-*O*-glucuronide at 40 and 80 μM were 0.053 and 0.070 pmol/min, respectively (Figure 1D). Western blotting results showed that the UGT1A9 protein was abundant in HeLa1A9 cells, whereas UGT1A9 was not expressed in wild-type HeLa cells (Figure 1E). BCRP, MRP1, MRP3, and MRP4 were detected in both wild-type HeLa and HeLa1A9 cells, whereas MRP2 was not detected (Figure 1E). It was noted that the wild-type and engineered HeLa1A9 cells had an identical pattern of transporter expression (Figure 1E). Taken together, these data suggested that the engineered HeLa1A9 cells strongly expressed active UGT1A9 protein.

### UGT1A9-Mediated Generation of Fraxetin-*O*-Glucuronides in HeLa1A9 Cells

**FIGURE 2:**
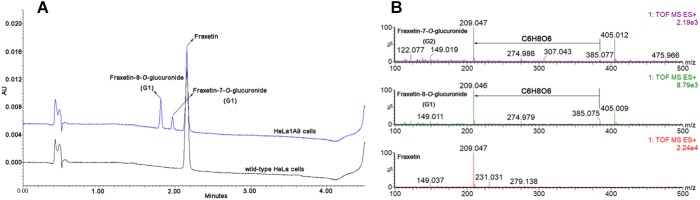
Ultra-high-performance liquid chromatography chromatograms and mass spectra of fraxetin and its related glucuronides. **(A)** UHPLC chromatograms of fraxetin, fraxetin-8-*O*-glucuronide (G1), and fraxetin-7-*O*-glucuronide (G2) generated in wild-type HeLa cells and HeLa1A9 cells. **(B)** electrospray ionization (ESI) mass spectra of fraxetin, G1, and G2 in positive ion mode.

After incubation of fraxetin with HeLa1A9 cells, two additional metabolites were generated (Figure 2A). By contrast, no metabolites were detected in wild-type HeLa cells. In addition, the two metabolites were 176.031 Da larger than fraxetin (Figure 2B), meaning that these two metabolites were two glucuronides. On the basis of another study (Xia et al., 2014), they were identified as fraxetin-8-*O*-glucuronide (G1) and fraxetin-7-*O*-glucuronide (G2), respectively.

### Kinetics of Fraxetin-*O*-Glucuronidation by UGT1A9 and in the HeLa1A9 Cell Lysate

**FIGURE 3:**
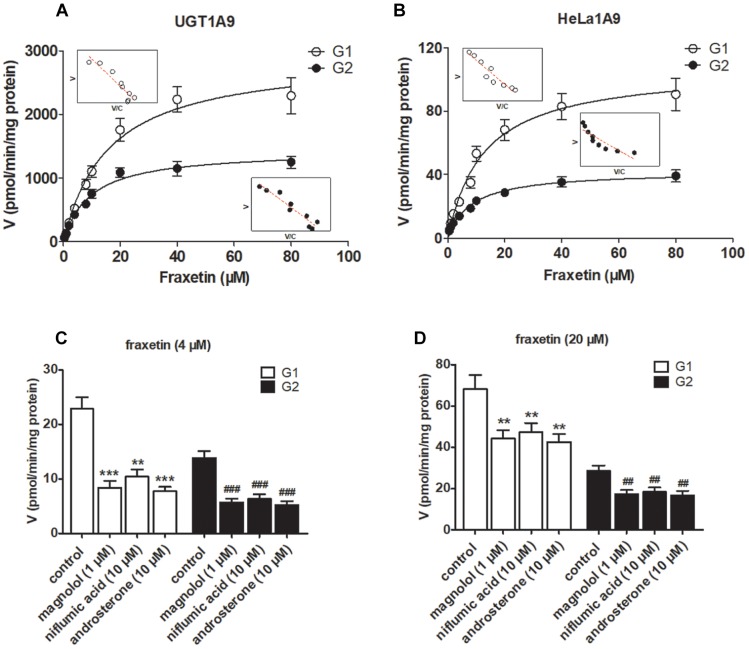
UGT1A9 catalyzes the glucuronidation of fraxetin. **(A)** Kinetic profiles for fraxetin-8-*O*-glucuronide (G1) and fraxetin-7-*O*-glucuronide (G2; 0.5–80 μM) generated by UGT1A9. **(B)** kinetic profiles for G1 and G2 (0.5–80 μM) generated by the HeLa1A9 cell lysate; in each panel, the inset shows the corresponding Eadie–Hofstee plot. **(C)** Inhibitory effects of magnolol (1 μM), niflumic acid (10 μM), and androsterone (10 μM) on the glucuronidation (formation of G1 and G2) at 4 μM of fraxetin. **(D)** Inhibitory effects of magnolol (1 μM), niflumic acid (10 μM), and androsterone (10 μM) on fraxetin-*O*-glucuronidation (formation of G1 and G2) at 20 μM. Data are expressed as mean ± SD. ^∗∗^,^##^*p* < 0.01, or ^∗∗∗^,^###^*p* < 0.001 as compared with the control.

The reaction kinetics for fraxetin-*O*-glucuronidation (G1 and G2) of recombinant UGT1A9 (Figure 3A) and of HeLa1A9 cell lysate (Figure 3B) were both modeled as the classical Michaelis–Menten equation. As shown in Table 1, G1 and G2 generated by UGT1A9 and HeLa1A9 cell lysate had similar *K_m_* values (∼8–16 μM), whereas *V_max_* and *CL*_int_ values exhibited significant differences (*p* < 0.001). The reason may be that UGT1A9 was much more concentrated in the recombinant material than in the HeLa1A9 cell lysate preparation.

To confirm the contribution of UGT1A9 to the generation of G1 and G2, chemical inhibitors were used to investigate the formation rates of G1 and G2. The results indicated that magnolol (1 μM), niflumic acid (10 μM), and androsterone (10 μM) significantly inhibited the formation of G1 and G2 at 4 μM (Figure 3C) and 20 μM (Figure 3D) of fraxetin, respectively. It was also noted that UGT1A9 was important for the glucuronidation of fraxetin.

### Concentration-Dependent Excretion of G1 and G2 From HeLa1A9 Cells

**FIGURE 4:**
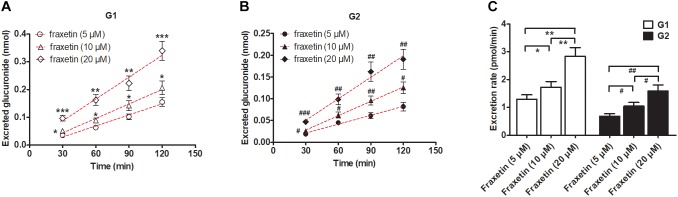
Concentration-dependent excretion of fraxetin-*O*-glucuronides in HeLa1A9 cells. **(A)** Excreted fraxetin-8-*O*-glucuronide (G1) in the extracellular solution at different concentrations of fraxetin (5, 10, and 20 μM). **(B)** excreted fraxetin-7-*O*-glucuronide (G2) during treatment with different concentrations of fraxetin (5, 10, and 20 μM). **(C)** excretion rates of G1 and G2 at different concentrations (5, 10, and 20 μM). Data are expressed as mean ± SD. ^∗,#^*p* < 0.05, ^∗∗,##^*p* < 0.01 or ^∗∗∗,###^*p* < 0.001 as compared with fraxetin at 5 or 10 μM.

In consideration of the *K*_m_ values of G1 and G2 production by the HeLa1A9 cell lysate (Table 1), three concentrations of fraxetin (5, 10, and 20 μM) were selected to evaluate the excretion rates of G1 and G2, respectively. Clearly, the excretion of G1 (Figure 4A) and G2 (Figure 4B) markedly increased with elevation of the concentration. In addition, the excretion rates of G1 and G2 at three concentrations had significant differences (*p* < 0.01; Figure 4C). Hence, fraxetin (10 μM) was chosen for the chemical and biological inhibition assays.

### Chemical Inhibition Assays Involving Transporter Inhibitors Ko143 and MK571

**FIGURE 5:**
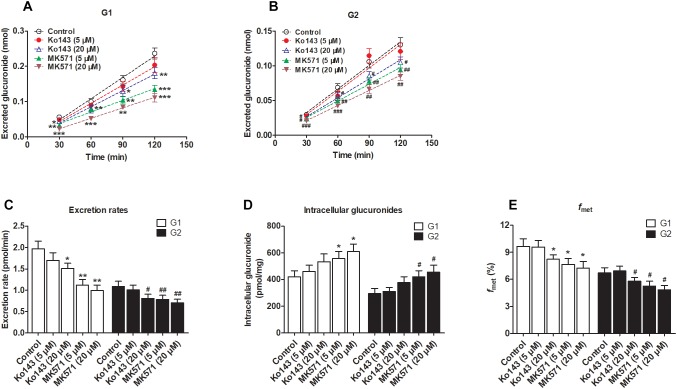
Effects of chemical inhibitors Ko143 (5 and 20 μM) and MK571 (5 and 20 μM) on the formation and efflux excretion of fraxetin-*O*-glucuronides in HeLa1A9 cells. **(A)** Effects of Ko143 (5 and 20 μM) and MK571 (5 and 20 μM) on the accumulation of fraxetin-8-*O*-glucuronide (G1) in the extracellular medium. **(B)** effects of Ko143 (5 and 20 μM) and MK571 (5 and 20 μM) on fraxetin-7-*O*-glucuronide (G2) excretion into extracellular solutions. **(C)** the influence of Ko143 (5 and 20 μM) and MK571 (5 and 20 μM) on the efflux excretion rates of G1 and G2. **(D)** effects of Ko143 (5 and 20 μM) and MK571 (5 and 20 μM) on the intracellular G1 and G2 levels. **(E)** the influence of Ko143 (5 and 20 μM) and MK571 (5 and 20 μM) on the fraction metabolized (*f*_met_) of fraxetin; Data are presented as mean ± SD. ^∗,#^*p* < 0.05, ^∗∗,##^*p* < 0.01, and ^∗∗∗,###^*p* < 0.001 as compared with the control.

We clearly saw that excreted glucuronides were significantly downregulated by the BCRP inhibitor Ko143 (20 μM; G1 by 29.4%, Figure 5A; G2 by 28.5%, Figure 5B). In contrast, during treatment with Ko143 (5 μM), the excretion of G1 and G2 showed almost no change (Figure 5A,B). The excretion rates (23.4% for G1 and 25.7% for G2, Figure 5C) and *f*_met_ (14.9% for G1 and 13.7% for G2, Figure 5E) values both significantly decreased, accompanied by no obvious alterations of intracellular glucuronides (27.0% for G1 and 28.2% for G2, Figure 5D) when Ko143 was 20 μM.

In addition, inhibition of MRPs by MK571 (5 and 20 μM) significantly altered the production and excretion of G1 (22.2–59.1%, Figure 5A) and G2 (19.6–42.4%, Figure 5B), respectively. Accordingly, the excretion rates were also remarkably decreased (43.1–49.4% for G1 and 27.5–34.8% for G2, Figure 5C). Besides, MK571 led to a significant increase in intracellular glucuronide levels (32.1–45.3% for G1 and 42.2–54.7% for G2, Figure 5D) as well as a reduction in fraction metabolized of fraxetin (21.1–25.3% for G1 and 21.7–28.1% for G2, Figure 5E). Taken together, these findings suggested that BCRP and MRPs both performed dominant functions in the excretion of fraxetin-*O*-glucuronides (G1 and G2).

### Effects of Ko143 and MK571 on Fraxetin-*O*-Glucuronidation Activities

**FIGURE 6:**
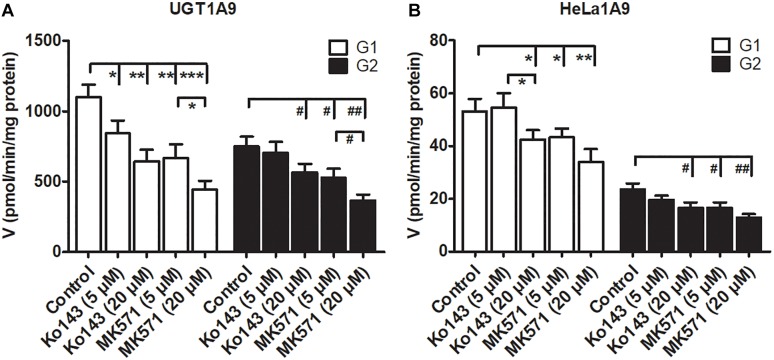
Chemical inhibitors Ko143 (5 and 20 μM) and MK571 (5 and 20 μM) exert inhibitory actions on fraxetin-*O*-glucuronidation activity of UGT1A9. **(A)** and of the HeLa1A9 cell lysate. **(B)**. Data are expressed as mean ± SD. ^∗,#^*p* < 0.05, ^∗∗,##^*p* < 0.01, and ^∗∗∗,###^*p* < 0.001 in comparison with the control.

Some studies have revealed that transporter inhibitors, Ko143 or MK571, have the potential to change the enzymatic activities of phase II enzymes (Quan et al., 2015; Qin et al., 2018a). To obtain a better interpretation of the chemical inhibition data on G1 and G2 excretion (Figure 5), we tested whether Ko143 and MK571 can modulate fraxetin-*O*-glucuronidation activities of UGT1A9 or of the HeLa1A9 cell lysate. As a result, Ko143 (20 μM) inhibited the fraxetin-*O*-glucuronidation by UGT1A9 (by 41.5% for G1 and by 24.9% for G2, Figure 6A) and by the HeLa1A9 cell lysate (by 20.4% for G1 and by 29.3% for G2, Figure 6B). Furthermore, MK571 at different concentrations (5 and 20 μM) significantly inhibited fraxetin-*O*-glucuronidation (by 18.7–59.7% for G1 and by 29.4–51.0% for G2) as shown in Figure 6A,B, respectively. These data also suggested that the efflux excretion of G1 and G2 (Figure 5) may be attributed to the significant inhibitory action of Ko143 and MK571 on fraxetin-*O*-glucuronidation (G1 and G2) activities (Figure 6).

### Effects of a Biological Knockdown of BCRP and Individual MRPs on Fraxetin-*O*-Glucuronidation in HeLa1A9 Cells

**FIGURE 7:**
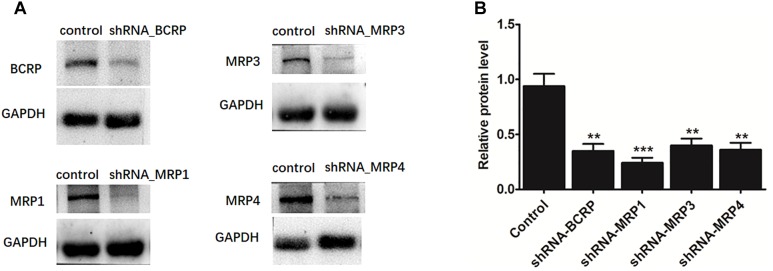
The protein expression **(A)** and efficiency of the knockdown **(B)** of BCRP, MRP1, MRP3, and MRP4 in HeLa1A9 cells after stable transfection of BCRP-shRNA, MRP1-shRNA, MRP3-shRNA, and MRP4-shRNA into HeLa1A9 cells, respectively. All the experiments were conducted in triplicate. ^∗^*p* < 0.05, ^∗∗^*p* < 0.01, and ^∗∗∗^*p* < 0.001 relative to the parameters of the control cells (scrambled shRNA transfection).

**FIGURE 8:**
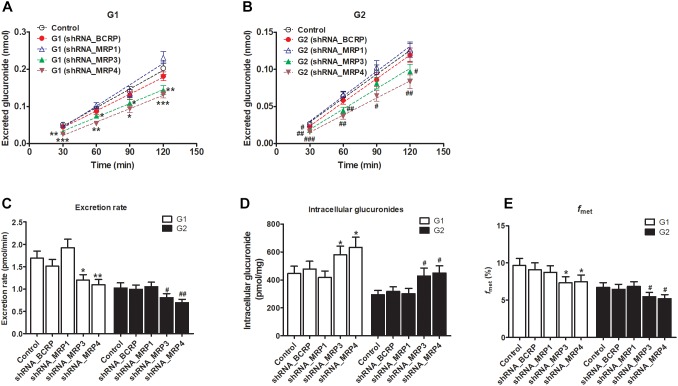
Effects of silencing of transporters BCRP and MRPs on the formation and excretion of fraxetin-*O*-glucuronides in HeLa1A9 cells. **(A)** The effects of partial silencing of the transporters on the accumulation of fraxetin-8-*O*-glucuronide (G1) in the extracellular medium. **(B)** the action of a knockdown of transporters on the excretion of fraxetin-7-*O*-glucuronide (G2) into the extracellular solution. **(C)** the influence of a knockdown of transporters on the excretion rates of G1 and G2. **(D)** effects of partial silencing of transporters on the intracellular G1 and G2 levels. **(E)** the influence of the knockdown of transporters on the total cellular glucuronidation (G1 and G2) of fraxetin. The concentration of fraxetin was 10 μM. Data are presented as mean ± SD. ^∗,#^*p* < 0.05, ^∗∗,##^*p* < 0.01, and ^∗∗∗,###^*p* < 0.001 as compared with the control.

The shRNA targeting BCRP, MRP1, MRP3, or MRP4 was transiently introduced into HeLa1A9 cells. Then, the protein expression (Figure 7A) and efficiency of the knockdown (Figure 7B) of transporters were verified and found to be ∼50% or lower after shRNA_BCRP and shRNA_MRP plasmids were transiently transfected into HeLa1A9 cells, consistent with our previous results (Quan et al., 2015; Sun et al., 2015; Qin et al., 2018a). Obviously, BCRP or MRP1 silencing did not cause any changes in excretion rates, intracellular glucuronides, and *f*_met_ values of fraxetin (Figure 8), suggesting that BCRP and MRP1 are not the important efflux transporters for the excretion of fraxetin-*O*-glucuronides.

The knockdown of MRP3 resulted in an obvious reduction in excreted glucuronides (by 19.8–39.4% for G1, Figure 8A; by 15.1–31.9% for G2, Figure 8B) and excretion rates (by 29.1% for G1 and by 21.1% for G2, Figure 8C) of the generated glucuronides, respectively. Intracellular glucuronide levels were significantly elevated after MRP3 silencing (by 30.2% for G1 and by 45.4% for G2, Figure 8D). In addition, the knockdown of MRP3 led to a moderate decrease in overall cellular fraxetin-*O*-glucuronidation (by 24.1% for G1 and by 18.6% for G2, Figure 8E). MRP4 silencing efficiency was similar to that of MRP3 silencing, including decreased excretion of G1 (by 33.6–51.1%, Figure 8A) and G2 (by 33.0–42.4%, Figure 8B), reduced excretion rates (by 34.8% for G1 and by 32.3% for G2, Figure 8C), elevated intracellular G1 and G2 (by 42.0% for G1 and by 52.4% for G2, Figure 8D) and decreased fraction metabolized of fraxetin (by 22.7% for G1 and by 23.1% for G2, Figure 8E). Taken together, these results illustrated that both MRP3 and MRP4 contributed greatly to the excretion of fraxetin-*O*-glucuronides (G1 and G2).

## Discussion

In this study, a previously developed HeLa1A9 cell model (Jiang et al., 2011) was applied to evaluate the efflux transport of the two glucuronides of fraxetin, a natural edible coumarin (Jyotshna et al., 2017). First, propofol (40 and 80 μM), a probe substrate of UGT1A9 (Hong et al., 2017), was tested to validate the functionality of the HeLa1A9 cell model (Figure 1). Second, the UGT1A9 protein was found to be abundant in HeLa1A9 cells, whereas UGT1A9 was not expressed in wild-type HeLa cells (Figure 1E). In addition, this model obviously catalyzed the glucuronidation of fraxetin via Michaelis–Menten kinetics (Figure 3) and mediated the efflux excretion of the two glucuronides in a concentration-dependent manner (Figure 4). Besides, assays of chemical inhibition by Ko143 and MK571 were carried out (Figure 5). The protein expression (Figure 7) of efflux transporters was verified and found to be ∼50% or lower after shRNA_BCRP and shRNA_MRPs plasmids were transiently transfected into HeLa1A9 cells, respectively. Biological inhibition experiments (Figure 8) illustrated that fraxetin-8-*O*-glucuronide and fraxetin-7-*O*-glucuronide were transported mainly by MRP3 and MRP4.

A chemical inhibitor, Ko143, is in widespread use for inhibition of transporter BCRP, whereas MK571 is considered a pan-MRP inhibitor (Quan et al., 2015; Qin et al., 2018a). Nonetheless, the effects of Ko143 and MK571 on fraxetin-*O*-glucuronidation should not be ignored. The reason is that Ko143 and MK571 can not only inhibit the function of BCRP and MRPs, respectively, but also change the glucuronidation activity of the UGT enzyme (Jiang et al., 2011; Quan et al., 2015; Qin et al., 2018a). Similar observations about modifications of glucuronidation activity were also noted in this study (Figure 7). Hence, the altered glucuronide excretion cannot be simply attributed to reduced BCRP or MRP activity. Notably, the glucuronidation activities modified by Ko143 and MK571 were limited confounding factors in the identification of the roles of BCRP and MRPs in the efflux excretion of glucuronides in chemical inhibition assays. In addition, although chemical inhibitors, dipyridamole and leukotriene C4, have been used to inhibit BCRP and MRP1/2, respectively, in other studies (Quan et al., 2015; Qin et al., 2018a), little is known about their inhibitory selectivity toward other transporters. Therefore, we did not perform supplementary experiments to investigate the roles of dipyridamole and leukotriene C4 in the excretion of fraxetin-*O*-glucuronides.

In addition, our results pointed toward important participation of MRP3 and MRP4 in the efflux excretion of fraxetin-*O*-glucuronides from the body. Traditionally, MRP3 is highly expressed in the liver (hepatocytes and choanocytes) and in enterocytes, as well as in the adrenal gland, kidneys, and the gallbladder (Ortiz et al., 1999; Van de Wetering et al., 2009), whereas MRP4 has been found in a variety of human tissues, with high levels of mRNA expression in the kidneys and prostate, and lower levels in the liver (Russel et al., 2008). After oral administration, fraxetin entered the enterocytes, passing across the intestinal wall into the blood stream, and later entering the liver and kidneys, where fraxetin conversion to fraxetin-*O*-glucuronides is mainly performed by the UGT1A9 enzyme. This is because a UGT1A9 isozyme was abundantly expressed in the liver and kidneys. Our results indicated that fraxetin-*O*-glucuronides in the liver may be subsequently transported via basolateral transporters MRP3 and MRP4 into the circulation (Figure 8). Nevertheless, the fraxetin-*O*-glucuronides formed in the kidneys may be excreted into urine through the kidney luminal apical transporter MRP4.

Here, a significant limitation is the unexplained role of MRP2 in glucuronide excretion in this HeLa1A9 cell model because MRP2 is not expressed in HeLa cells (Quan et al., 2015). Thus, the function of MRP2 in the excretion of fraxetin-*O*-glucuronides remains unknown. Traditionally, BCRP and MRP2 mediate the transport of glucuronides to bile (Quan et al., 2015; Wang M. et al., 2016; Qin et al., 2018a), and the biliary excretion of phase II conjugates is severely impaired in BCRP or MRP2-deficient rats (Zamek-Gliszczynski, 2006; Zamek-Gliszczynski et al., 2011). In this study, it was proved that fraxetin-*O*-glucuronides are not transported via the canalicular transporter BCRP (Figure 8). Therefore, the elucidation of transporter MRP2’s involvement in the excretion of fraxetin-*O*-glucuronides would help to understand the biliary clearance of fraxetin, which may also better explain the pharmacokinetics of fraxetin (Wang H. et al., 2016; Wang et al., 2017). Recently, a newly developed MDCKII-MRP2-UGT1A1 cell model was extensively employed to characterize how overexpression of MRP2 and UGT1A1 affects the cellular kinetics of a flavonoid glucuronidation processes (Wang M. et al., 2016). This cell line provides a practical approach to further evaluation of MRP2 function in the excretion of fraxetin-*O*-glucuronides.

Efflux transporter-mediated excretion of glucuronide conjugates has been proved to be a key step in the overall glucuronidation activity of a cell or tissue (Quan et al., 2015; Sun et al., 2015; Qin et al., 2018a). These data additionally implied that not only UGT activity but also MRP3 or MRP4 transport capacity of cells influenced the glucuronidation rates. Besides, the interplay between specific UGT isoforms and relevant efflux transporters, namely the glucuronidation–efflux interplay, can also affect the cellular glucuronidation of fraxetin, further limiting the oral bioavailability of drugs (Sun et al., 2015). This is because glucuronides can be hydrolyzed back to aglycone by the action of β-glucuronidases (Quan et al., 2015; Sun et al., 2015; Qin et al., 2018a). The ability to alter the systemic exposure to glucuronides means that it is possible to change the systemic exposure to aglycones, which are usually pharmacologically more active. Therefore, it was assumed that the understanding of how the efflux transporter expression level can affect glucuronide formation and excretion should allow us to find a way to increase the bioavailability of compounds susceptible to extensive glucuronidation.

On the other hand, drugs are often ingested concomitantly with fraxetin-containing foods or herbal medicines; thus, there is a possibility for phytochemical-mediated food–drug interactions, especially in the gut where concentrations are the highest (Sjöstedt et al., 2016). In addition, the excitatory or inhibitory effects of fraxetin on drug-metabolizing enzymes or transporters may not only trigger adverse clinical herb–drug interactions but also result in disorders of the metabolism of endogenous substances (Yao et al., 2017). This is because several signal transduction molecules including estradiol, estrone, bile salts, cAMP are the endogenous substrates of UGTs and transporters, which maintain the balance of physiological function (International Transporter Consortium, 2010; Yang et al., 2017). Hence, the effects of fraxetin on enzymes or transporters seem to be especially important for avoidance of unnecessary interactions between fraxetin (or fraxetin-containing foods, herbs, or drugs) and endogenous substances. So far, this issue seems to be only a minor concern for the interactions because of the absence of an inhibitory action of fraxetin on transporters BCRP and MRP2 (Sjöstedt et al., 2016). Unfortunately, the inhibitory effects of fraxetin on human CYPs, UGTs, and other transporters (e.g., MRP1, MRP3, and MRP4) all remain unclear, introducing some uncertainties into the interactions. Therefore, the stimulatory or inhibitory effects of fraxetin on drug-metabolizing enzymes and other transporters needed to be explored in-depth.

Moreover, genetic polymorphisms among different ethnic groups are important factors affecting the glucuronidation or efflux excretion of drugs and cannot be ignored (Lévesque et al., 2007). In the clinic, before oral administration of therapeutic drugs (e.g., mycophenolic acid, anthracyclines, or methotrexate), polymorphisms of enzymes or transporters in individual subjects should be tested to determine an appropriate dose to ensure plasma concentrations of the drugs within the therapeutic window (Lévesque et al., 2007; Bruhn and Cascorbi, 2014; Tanaka et al., 2014). For instance, compared to controls, mycophenolic acid exposure is significantly lower for UGT1A9-275/-2152 carriers, and UGT1A9^∗^3 carriers experience greater exposure to mycophenolic acid and its related acyl glucuronides (Lévesque et al., 2007). Similarly, the MRP3 189A > T regulatory polymorphism appears to be associated with altered hepatic MRP3 mRNA expression (Bruhn and Cascorbi, 2014), whereas patients with a homozygous variant allele in MRP4 G2269A, C912A, or G559T require high frequency of 6-mercaptopurine dose reduction as compared with non-homozygous individuals (Tanaka et al., 2014). Therefore, humans with UGT1A9 enzyme dysfunction or genetic polymorphisms of MRP3 and MRP4 most often show alterations in the metabolic routes for fraxetin, and possibly even toward toxic pathways. In this study, the assays mainly focused on the functional evaluations of wild-type UGT1A9, BCRP, and MRPs. This part of the pathway is a rate-limiting step for individuals with genetic mutations of UGT1A9 or efflux transporters.

## Conclusion

In conclusion, HeLa1A9 cells were stably transfected with the *UGT1A9* gene (Figure 1E). Meanwhile, BCRP, MRP1, MRP3, and MRP4 were all detected in both wild-type HeLa and HeLa1A9 cells, whereas the MRP2 protein was not detected (Figure 1E). In addition, the current variant HeLa1A9 cells were fully active toward propofol (the specific substrate of UGT1A9) (Figure 1) and fraxetin (Figure 3), and in pumping out propofol-*O*-glucuronide (Figure 1) and fraxetin-*O*-glucuronides (Figure 4) in a concentration-dependent manner. Moreover, we showed that the cellular excretion of fraxetin-*O*-glucuronides was potentially mediated mainly by MRP3 and MRP4 according to chemical inhibition experiments (Figure 5) and shRNA-mediated silencing (Figure 8). These results should help to clarify the metabolism and disposal of fraxetin.

## Author Contributions

ZQ, BZ, XZ, and ZY designed the research. ZQ, BZ, SL, and JX conducted and performed the majority of the experiments. JY, BZ, SL, and JX assisted and supported several experiments and dealt with the statistical analysis. ZY, XZ, FG, and XY supervised the research and revised the manuscript. All authors approved the final version of the manuscript to be published.

## Conflict of Interest Statement

The authors declare that the research was conducted in the absence of any commercial or financial relationships that could be construed as a potential conflict of interest. The reviewer BW declared a shared affiliation, though no other collaboration, with several of the authors, SL, JX, ZY, XY, to the handling Editor.
